# Editorial: Current developments in artificial organs and engineered* ex-situ* perfused organs

**DOI:** 10.3389/ti.2026.16911

**Published:** 2026-06-09

**Authors:** Cristiano Amarelli, Jutta Arens, Cécile Legallais, Thierry Berney

**Affiliations:** 1 Department of Cardiac Surgery and Transplants, Monaldi, Azienda Ospedaliera dei Colli, Naples, Italy; 2 Department of Biomechanical Engineering, University of Twente, Enschede, Netherlands; 3 Université de technologie de Compiègne, CNRS, BMBI (Biomechanics and Bioengineering), Compiègne, France; 4 Department of Nephrology, Transplantation and Clinical Immunology, Hospices Civils de Lyon, Groupement Hospitalier Centre, Lyon, France; 5 Faculty of Natural Sciences and Medicine, Ilia State University, Tbilisi, Georgia

**Keywords:** artificial organs, machine perfusion, hypothermic, normothermic, reconditioning

## Introduction

Despite the steady increase in solid organ transplant activity worldwide, a substantial and persistent mismatch remains between the growing prevalence of end-stage organ failure and the limited availability of donated organs. The unmet needs extend well beyond the population of wait-listed and transplanted patients [1], considering that less than 10% of the global needs for transplantation are currently met. Reconditioning of discarded organs, artificial and bioartificial solutions, and xenotransplantation have been proposed for replacing almost every failed organ.

The recent rise of *ex vivo* machine perfusion (EVMP) has provided an opportunity to recondition marginal-quality organs and prevent their discard. This technology also opens a window of opportunity for intervention to assess or repair the organ’s function during the perfusion period (for example, using gene therapy strategies) and even to reprogram organs failing due to inherited diseases [2].

This special issue was designed to collect review and original articles addressing current developments in artificial organ development and *ex situ* organ perfusion (heart, lung, kidney, or liver) for various purposes: transportation, assessment, reconditioning, repair, and research model development.

As machine perfusion is already established as a method for reconditioning kidneys and livers [3–5], it is no surprise that a vast majority of submitted and published articles came from the field of thoracic organ transplantation [6, 7], where the unmet needs are greater.

## The value of machine perfusion

A review from Lund University covers the efforts made to expand the donor pool including bioengineering techniques and the use of *ex vivo* graft perfusion (Niroomand et al.). It also covers modifications and treatments that have been tested in clinical trials, in addition to research efforts in both abdominal and thoracic organs. Overall, this article discusses recent innovations in machine perfusion, but organ bioengineering advances are discussed extensively, reviewing how these strategies can improve and increase the quality of donor organs. The main focus is on the lung and the heart, but abdominal organs (liver, kidney, pancreas) are also covered to provide an updated and broad review.

EVMP directly addresses ischemia–reperfusion injury (IRI) and primary graft dysfunction (PGD), which represent the principal barriers to the wider adoption of DCD hearts. The extensive review by Tessari et al. from the University of Padova describes a large number of animal models of EVMP in the context of DCD heart transplantation (Tessari et al.), highlighting the crucial role of preclinical research in the development of the field.

As for the need for pre-clinical models, system miniaturization and single-organ study design can simultaneously enhance experimental rigor, ethical compliance, and translational relevance in *ex situ* lung perfusion research. The group from the University of Hannover developed a low-volume EVMP model in the rat for double- and single-lung application, enabling cost-effective optimal compliance “reduction” of the 3R principles of animal research (Replacement, Reduction, and Refinement) (Katsirntaki et al.). Most relevant perfusion parameters confirmed the model’s validity, with homogeneous lung perfusion evidenced by uniform lung surface temperature, and histological examination confirmed intact lung architecture without infarcts or hemorrhages.

The group at the University of Bari is one of the frontline centers striving to expand the heart donor pool using reconditioning strategies. They have reviewed use of veno-arterial extracorporeal membrane oxygenation (VA ECMO) to ensure organ perfusion and provide the transplanted heart with adequate rest while recovering from IR (Giovannico et al.). The review describes how mechanical circulatory support may turn severe PGD into a reversible condition. They state that, in the new era of heart transplantation, early use of VA-ECMO increases patient survival.

## Assessment of the graft during EVMP

One of the added values of EVMP has been the possibility to assess graft function, thereby predicting graft outcome and protecting the recipient from the risk of unpredictable events after transplantation [8]. (Van der Hoek et al.) propose that integrating medical imaging into *ex vivo* human-sized organ perfusion fundamentally enhances organ condition assessment by providing both spatially resolved and functional insights that complement traditional viability markers. The systematic review from the University of Twente shows that medical imaging is a powerful tool to assess organ condition in perfused hearts, kidneys, and livers. Laser speckle contrast imaging, ultrasound, computed tomography, and magnetic resonance imaging have been convincingly used to identify local ischemic regions and quantify intra-organ perfusion. New imaging techniques, such as ^31^P magnetic resonance spectroscopy and near-infrared spectroscopy, may enable detailed investigation of the graft’s metabolic activity. Scalability and imaging costs for organ assessment remain major concerns for clinical adoption.

An original study from Erasmus University, in a porcine model of normothermic heart EVMP, proposes a novel approach using electrophysiological (EP) parameters derived from electrical mapping as as an alternative to biomarkers for assessing post-ischemic cardiac performance (Amesz et al.). This technique offers an objective adjunct (and potential improvement) over the criticized lactate-based DCD heart assessment. Potential voltages, slopes, and conduction velocities measured on the left and right ventricles were computed from unipolar electrograms and compared between groups. Potential voltages and slopes were decreased in hearts with severe warm ischemia. The study concludes that EP parameters could aid in decision-making about the transplantability of DCD hearts.

The University of Groningen team demonstrated in an ovine proof-of-concept that pressure–volume (PV) loop analysis can be used during normothermic ESHP to functionally assess left-ventricular (LV) performance (Ertugrul et al.). This technique is a further attempt to move beyond the reliance on biochemical markers to assess perfusion. This method uses a physiological “working-mode” test to quantify LV function through end-systolic elastance (ESE) measured at 60 and 120 min. The system allows adjustment of preload and afterload to the left ventricle. By increasing preload and measuring ESE, left ventricular function can be reliably assessed, supporting better graft selection for both DBD and DCD donor hearts.

A systematic review of non-randomized controlled trials on patients undergoing transplantation with reconditioned lungs via EVMP was published by the University of Torino, aiming to assess the association between levels of proinflammatory biomarkers (adhesion molecules, chemokines, cytokines, damage-associated-molecular-patterns, growth-factors, metabolites) during EVMP and development of grade 3 PGD within the first 72 h post-transplant (Costamagna et al.). Meta-analysis revealed that the chemokine panel has the strongest association rather than relying solely on traditional physiologic/biochemical monitoring. Pooled chemokine panels measured at the beginning or end of the perfusion period are associated with the development of grade 3 PGD within the first 72 h after lung transplantation, supporting biomarker-guided risk stratification during EVMP.

An interesting protocol for a paired-lung pilot study is presented by two organ donor centers from Spain (Grando et al.). It will include seven organ donors after brain death or after controlled cardiac death. The left lung will be preserved in static cold storage (SCS) and the right lung will be perfused with normothermic EVMP. Samples from bronchoalveolar lavage, perfusion and preservation solutions, and lung biopsies will be collected from both lungs. The study is designed to test whether EVMP reshapes the lung microbiome, and whether microbiome shifts align with changes in local inflammatory activation.

The University of Innsbruck reports on a study evaluating mitochondrial function by high-resolution respirometry as a viability assessment of liver grafts (Hofmann et al.). They observed that in livers taken off normothermic EVMP for diagnostic biopsy and preserved in SCS conditions thereafter, mitochondrial respiration was stable up to 4 h but decreased afterwards. They conclude that SCS can be safely applied to extend the biopsy measurement window for up to 4 h to determine organ quality, and that human liver respiration degrades beyond 4 h of SCS following normothermic machine perfusion.

## Intervention to improve graft quality during EVMP

EVMP not only allows the reconditioning of marginal grafts but also provides a window of opportunity for various types of interventions to further improve graft quality. The combination of intervention and assessment methods during EVMP is a key toward maximizing marginal donor organ utilization.

Dawn Bowles’ lab at Duke University delivered a muscle-tropic recombinant adeno-associated virus (AAV), AAV-SLB101, to porcine hearts during a brief normothermic EVMP period (Dewan et al.). *Ex situ* normothermic heart perfusion can serve as a scalable “gene-delivery window” to transduce a donor heart with a muscle-tropic AAV vector, achieving early-onset, durable myocardial transgene expression after transplant that lasts up to 120 days, with minimal off-target transgene expression. The authors propose that this exploratory study will serve as a critical foundation for translational studies using therapeutic transgenes to improve outcomes in heart transplantation.

Boffini et al. propose that EVMP outcomes may be limited by gravity-dependent fluid accumulation and ventilation–perfusion (V/Q) mismatch in the supine position—particularly in the dorsal lung regions (Boffini et al.). They designed a tilting dome that allows the lung block to be progressively moved from 0° to 90° during perfusion, aiming to maintain physiologic function while potentially reducing dorsal congestion. This feasibility study, conducted in a porcine model without a control group, showed that the lungs maintained satisfactory gas-exchange parameters throughout the 4-h experimental period. Histology showed neither edema nor congestion; granulocytic infiltration was sporadic in the lung interstitium and absent from the alveoli; no hemorrhage or microthrombosis was detected.

A group from Astana has studied the impact of VA ECMO, including bronchodilator administration, hemofiltration, and cytokine removal using a Cytosorb membrane, on lung preservation in a porcine model (Faizov et al.). In their study, ECMO and hemofiltration maintained lung function for at least 24 h, as supported by a study from the Bartlett group a few years ago [9]. Additional cytokine removal significantly improved organ quality over 24 h of EVMP.

## A look at vascular composite allografts (VCA)

One of the frontiers of EVMP is the potential to expand its use to VCA transplantation [10], including for the preservation of uterine grafts [11]. The scarcity of literature reflects the relatively low level of clinical activity in this field and the need for clear targets to define its effectiveness.

A study from Harvard Medical School reported the impact of subnormothermic EVMP on prolonged preservation of VCA tissues in a porcine hind limb allotransplantation model. (Goutard et al.) demonstrate that oxygenated, acellular subnormothermic machine perfusion can preserve VCAs for 24 h and still support successful allotransplantation, whereas time-matched SCS leads to severe post-transplant muscle degeneration and early failure. This preclinical evidence provides a strong translational proof of principle that EVMP can meaningfully extend the VCA preservation window.

Finally, a group from Aachen has developed an artificial womb (Heyer et al.). This artificial organ is not designed as an implantable device, but as an *ex vivo* “bridge-to-life” for severely prematurely born infants. This extremely interesting paper lacks *in vivo* data but describes the performance achieved in adapting to the physiologic growth of an extremely preterm neonate over a 4-week period, envisioning its future possible adoption. Results of the *in vitro* tests showed a temperature constancy of 36.8 °C without pressure loss. A filtration and disinfection system was designed and has proven strong disinfection capabilities, effective filtering of metabolic waste, and the ability to avoid phospholipid washout.

## Conclusion

This collection of articles demonstrates the vitality and ongoing improvements in the field of organ reconditioning, repair, and replacement using advanced technologies. Research on EVMP is progressing rapidly, especially for thoracic organs, and is entering the field of VCA, but it also offers an impressive source of pathophysiological insights. So far, technical challenges have prevented the clinical application of EVMP to the pancreas on a convincing basis, but research is ongoing [12], as graft preservation remains a main focus for reshaping the field. These strategies are only one of the avenues studied, developed, and implemented to narrow the ever-increasing gap between the number of quality donor organs and the number of patients living with end-stage organ failure and on the waiting list for a kidney, liver, heart, or lung.

## References

[1] Oniscu G Muller E Wilson C Horton R ESOT–Lancet Commission on Transplantation. The ESOT–lancet commission on transplantation: a new vision for global sustainability, innovation, and equity in organ transplantation. The Lancet (2025) 406:313–5. 10.1016/S0140-6736(25)01197-3 40614742

[2] Iske J Schroeter A Knoedler S Nazari-Shafti TZ Wert L Roesel MJ Pushing the boundaries of innovation: the potential of *ex vivo* organ perfusion from an interdisciplinary point of view. Front Cardiovasc Med (2023) 10:1272945. 10.3389/fcvm.2023.1272945 37900569 PMC10602690

[3] Oki R Rocha I Al-Juburi S Rajendran L Kerby E Mohamed A The individual impact of machine perfusion on liver and kidney on donor expansion in simultaneous liver and kidney transplantation. Transpl Int (2025) 38:14807. 10.3389/ti.2025.14807 40994627 PMC12454159

[4] Jaynes CL Goggins WC Holzner ML Garonzik-Wang J Leuvenink HGD . No kidney left behind: rescuing unused donor kidneys for transplant at the first centralized assessment and repair center. Transpl Int (2025) 38:15424. 10.3389/ti.2025.15424 41394518 PMC12698490

[5] Darius T Jochmans I Foguenne M Hoste E Randon C Bracke B Nationwide hypothermic machine perfusion for ECD and DCD kidney transplantation in Belgium: one-year outcomes and impact on transplant rates and budget impact analysis. Transpl Int (2025) 38:15282. 10.3389/ti.2025.15282 41323959 PMC12657249

[6] Amarelli C Bello I Aigner C Berman M Boffini M Clark S European society of organ transplantation (ESOT) consensus statement on machine perfusion in cardiothoracic transplant. Transpl Int (2024) 37:13112. 10.3389/ti.2024.13112 39649067 PMC11620879

[7] Schiavon M Bennett D Boffini M Carillo C Dell’Amore A Fumagalli J Use of normothermic perfusion machines in lung transplantation: consensus statement of the Italian society of organ and tissues transplantation group with DELPHI method. Transpl Int (2025) 38:14762. 10.3389/ti.2025.14762 41064162 PMC12500477

[8] Sponga S Vendramin I Salman J Ferrara V De Manna ND Lechiancole A Heart transplantation in high-risk recipients employing donor marginal grafts preserved with *Ex-Vivo* perfusion. Transpl Int (2023) 36:11089. 10.3389/ti.2023.11089 37547752 PMC10401590

[9] Johnson MD Fallon BP Langley M Kayden A Shenton H Schneider B Prolonged (24-hour) normothermic *ex vivo* heart perfusion facilitated by perfusate hemofiltration. ASAIO J (2022) 68(10):1282–9. 10.1097/MAT.0000000000001649 36194099

[10] Chakradhar A Yu M Talbot SG . Systematic review of emerging technologies in vascularized composite allotransplantation. Front Transpl (2026) 5:1760147. 10.3389/frtra.2026.1760147 41878305 PMC13006663

[11] Drivas EM Khaki S Loftin AH Lamsehchi N Johannesson L Oh BC A comprehensive review of *Ex-Vivo* machine perfusion in uterus transplantation. Transpl Int (2025) 38:15254. 10.3389/ti.2025.15254 41439262 PMC12719308

[12] Ferrer-Fàbrega J Mesnard B Messner F Doppenberg JB Drachenberg C Engelse MA European society for organ transplantation (ESOT) consensus statement on the role of pancreas machine perfusion to increase the donor pool for beta cell replacement therapy. Transpl Int (2023) 36:11374. 10.3389/ti.2023.11374 37547751 PMC10402633

